# Counteracting Quasispecies Adaptability: Extinction of a Ribavirin-Resistant Virus Mutant by an Alternative Mutagenic Treatment

**DOI:** 10.1371/journal.pone.0005554

**Published:** 2009-05-14

**Authors:** Celia Perales, Rubén Agudo, Esteban Domingo

**Affiliations:** 1 Centro de Biología Molecular “Severo Ochoa” (CSIC-UAM), Consejo Superior de Investigaciones Científicas (CSIC), Campus de Cantoblanco, Madrid, Spain; 2 Centro de Investigación Biomédica en Red de Enfermedades Hepáticas y Digestivas (CIBERehd), Barcelona, Spain; Institut Pasteur Korea, Republic of Korea

## Abstract

**Background:**

Lethal mutagenesis, or virus extinction promoted by mutagen-induced elevation of mutation rates of viruses, may meet with the problem of selection of mutagen-resistant variants, as extensively documented for standard, non-mutagenic antiviral inhibitors. Previously, we characterized a mutant of foot-and-mouth disease virus that included in its RNA-dependent RNA polymerase replacement M296I that decreased the sensitivity of the virus to the mutagenic nucleoside analogue ribavirin.

**Methodology and Principal Findings:**

Replacement M296I in the viral polymerase impedes the extinction of the mutant foot-and-mouth disease virus by elevated concentrations of ribavirin. In contrast, wild type virus was extinguished by the same ribavirin treatment and, interestingly, no mutants resistant to ribavirin were selected from the wild type populations. Decreases of infectivity and viral load of the ribavirin-resistant M296I mutant were attained with a combination of the mutagen 5-fluorouracil and the non-mutagenic inhibitor guanidine hydrocloride. However, extinction was achieved with a sequential treatment, first with ribavirin, and then with a minimal dose of 5-fluorouracil in combination with guanidine hydrochloride. Both, wild type and ribavirin-resistant mutant M296I exhibited equal sensitivity to this combination, indicating that replacement M296I in the polymerase did not confer a significant cross-resistance to 5-fluorouracil. We discuss these results in relation to antiviral designs based on lethal mutagenesis.

**Conclusions:**

(i) When dominant in the population, a mutation that confers partial resistance to a mutagenic agent can jeopardize virus extinction by elevated doses of the same mutagen. (ii) A wild type virus, subjected to identical high mutagenic treatment, need not select a mutagen-resistant variant, and the population can be extinguished. (iii) Extinction of the mutagen-resistant variant can be achieved by a sequential treatment of a high dose of the same mutagen, followed by a combination of another mutagen with an antiviral inhibitor.

## Introduction

RNA virus genomes do not replicate as a defined nucleotide sequence, but as complex and dynamic mutant spectra termed viral quasispecies (reviews in [1], [2]). Quasispecies dynamics is the result of high mutability, and constitutes a major factor in the adaptability of RNA viruses to changing environments. From a practical point of view, adaptability represents a challenge for the design of effective treatments to control diseases associated with highly variable viruses [3]–[10]. In particular, viral mutants resistant to antiviral inhibitors are systematically selected both in experimental settings and in clinical practice. New antiviral strategies are now under investigation to avoid or minimize selection of drug-resistant virus mutants, and the ensuing treatment failure. One such strategies is termed lethal mutagenesis, that aims at extinguishing virus through an increase of the virus mutation rate, above the maximum level compatible with virus viability [2], [11]–[14]. An important development in lethal mutagenesis was the discovery that the nucleoside analogue ribavirin [1-(β-D-ribofuranosyl)-1*H*-1,2,4-triazole-3-carboxamide] (R), currently licensed as an antiviral agent, is mutagenic for poliovirus [15]–[17]. A mutagenic activity of R was subsequently reported for other viruses, including foot-and-mouth disease virus (FMDV) and hepatitis C virus (HCV) [18]–[25]. R has several mechanisms of action [26], [27], and a relevant, still unsettled question is whether R acts as a mutagenic agent in the course of treatment of HCV-infected patients, when R is administered in combination with IFN-α [28]–[33].

A critical issue in a lethal mutagenesis approach to treat viral infections is whether mutagen-resistant mutants can be selected, and whether they can jeopardize treatment efficacy. R-resistant mutants of several RNA viruses have been selected and characterized [34]–[38]. A poliovirus (PV) mutant resistant to R included replacement G64S in its RNA-dependent RNA polymerase (RdRp, termed 3D), and this replacement increased template-copying fidelity, and rendered PV less adaptable to a complex environment, relative to the wild type virus [39]–[43]. Despite the PV and foot-and-mouth disease virus (FMDV) polymerases being closely related structurally [44], a FMDV mutant with decreased sensitivity to R included replacement M296I in its 3D, a site distant from that where the PV substitution G64S is located. The FMDV mutant is termed M296I, and, contrary to substitution G64S in PV, M296I increased modestly the A→G transition frequency relative to wild type 3D, and restricted specifically the incorporation of R-triphosphate (RTP) during RNA elongation [37], [45]. The comparison of PV mutant G64S and FMDV mutant M296I has documented that different sites in the picornavirus polymerase can modulate incorporation of nucleotide analogues, and that R-resistance does not require a general increase of template-copying fidelity of the polymerase [45]. Lethal mutagenesis of FMDV has been studied in our laboratory, with regard to the effect of viral load and viral fitness on extinction [46], changes in population complexity associated with extinction [18], [47], and the advantages of a combination of a mutagen and antiviral inhibitors over a mutagen alone as a treatment to achieve virus extinction in cell culture [48], [49]. The effect of substitution M296I in 3D on FMDV extinction has not been studied.

Here we report that FMDV mutant M296I, with no other mutations in its genome, is not extinguished upon passage in the presence of high R concentrations. In contrast, the wild type (WT) virus was extinguished under the same treatment and passage conditions, and no R-resistant mutants were selected under high R concentrations. However, extinction of FMDV M296I was achieved by treatment with another mutagen (5-fluorouracil, FU), in combination with an inhibitor of viral replication [guanidine hydrochloride, GU [49]–[51]]. Implications for antiviral designs based on lethal mutagenesis are discussed.

## Results

### Replacement M296I in 3D can prevent FMDV extinction by high ribavirin concentrations

**Figure 1:**
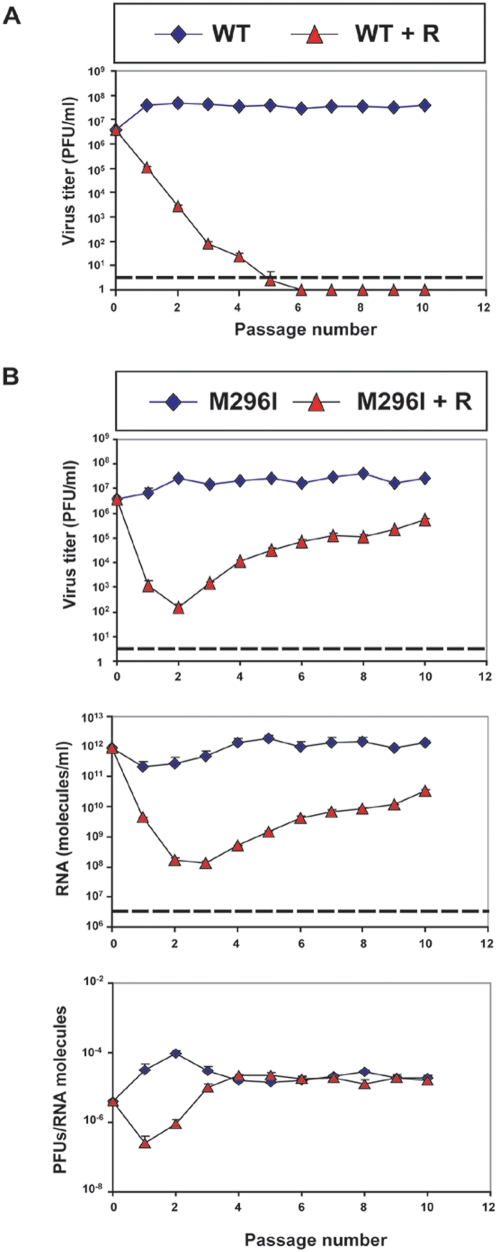
Absence of extinction of FMDV M296I after treatment with ribavirin. BHK-21 cells (2×10^6^ cells) were initially infected with the indicated viruses (WT, wild type FMDV; M296I, FMDV with substitution M296I in 3D) at a multiplicity of infection (MOI) of about 0.3 PFU/cell. In succesive passages, the same number of cells were infected with the supernatant from the previous passage. Viruses and the absence or presence of ribavirin (R) (5 mM) are indicated in the boxes at the top of panels A and B; (A) Infectivity of FMDV WT in the cell culture supernatants. Values are the mean±SD (error bars) of triplicate determinations. The discontinuous line indicates the limit of detection of FMDV infectivity. (B) Infectivity, viral RNA levels and specific infectivity in the supernatants of BHK-21 cells infected with FMDV M296I. Titrations and quantifications of FMDV RNA were carried out in triplicate; standard deviations are given. Viral RNA was extracted and quantified by real time PCR. The discontinuous lines indicate the limit of detection of infectivity and viral RNA. Values of specific infectivity (PFUs/RNA molecules) were calculated from the infectivities and viral RNA levels given in the two previous panels of (B). Procedures for titration of virus infectivity and quantification of FMDV RNA, as well as the positive and negative controls included in the assays, are described in Materials and Methods.

Serial cytolytic passages of FMDV in the presence of increasing concentrations of R (200 µM and up to 800 µM) resulted in selection of a FMDV mutant harboring amino acid substitution M296I in the polymerase (3D) [37]. The mutant FMDV displayed higher replicative fitness than the wild type FMDV in the presence of R, but lower fitness in the absence of R. In polymerization assays *in vitro*, the mutant 3D showed decreased capacity to use RTP as substrate (in the place of GTP and ATP) than wild type 3D [37], [45]. To investigate whether substitution M296I in 3D could jeopardize the extinction of FMDV by ribavirin treatment, virus that differed only at amino acid residue 296 in 3D were expressed from plasmids pMT28-WT, and pMT28-M296I, respectively. Throughout the present report, these two progeny viruses are abbreviated as WT and M296I, respectively. FMDV WT and M296I were subjected to serial cytolytic passages in BHK-21 cells in the presence or absence of a high concentration (5 mM) of R. FMDV WT was extinguished by passage 6, according to the criteria for virus extinction (absence of infectivity and of virus-specific genetic material), detailed in Materials and Methods (Fig. 1A). In contrast, FMDV M296I was not extinguished, and, beginning at passage 3, the virus increased progressively its replication rate (Fig. 1B). The evolution of infectivity paralleled that of viral RNA level (viral load), and the specific infectivity in the presence and absence of R remained constant form passage 4 until at least passage 10. The mutant population that increased its replication in the presence of R (Fig. 1B) was analyzed by comparing the consensus nucleotide (and deduced amino acid sequence) of the entire viral genome at passages 2, 5, 8 and 10. The results (Table 1) show an increase in the number of mutations that reached 14 (4 nonsynonymous and 10 synonymous, of which 2 were not dominant in the consensus) at passage 10. This represents a rate of accumulation of mutations of 1.5 per passage, a rate 6- to 15-fold higher than estimated for large population passages of FMDV in the absence of R [52], [53] (p< 0.005; χ^2^ test; calculated from the number of mutations in large populations passages in the absence and presence of R). G→A and C→U transitions, those induced by R [37], [45], amounted to 50% of the total (Table 1). Substitution M296I in 3D was maintained, and additional nonsynomymous mutations in VP3, 2C, 3A and 3D became dominant. Because these mutations are likely to confer a selective advantage to the virus in the presence of R, they are currently under study. Although the presence of substitution M296I in 3D at the onset of R treatment prevented extinction of FMDV during cytolytic infections, this mutation was not selected when FMDV WT clone was subjected to treatment with high R concentrations, and FMDV WT was extinguished.

**Table 1:** Mutations in FMDV M296I passaged in the presence of ribavirin.

Genomic region^b^	Passage 2		Passage 5		Passage 8		Passage 10	
	Mutation^c^	Amino acid^c^	Mutation^c^	Amino acid^c^	Mutation^c^	Amino acid^c^	Mutation^c^	Amino acid^c^
5′ UTR					A809G	-	A809G	-
5′ UTR	U986C	-	U986C	-	U986C	-		
L					U1044C	-	U1044C	-
VP2							C2379U/C	-
							C2625U/C	-
**VP3**					**A3112G**	**I188V**	**A3112G**	**I188V**
					C3144U	-	C3144U	-
VP1					U3699C	-	U3699C	-
2B	A4131C	-	A4131C	-	A4131C	-		
					C4134C/U		C4134U	-
**2C**					**C4748U**	**T135I**	**C4748U**	**T135I**
**3A**					**G5602A**	**D102N**	**G5602A**	**D102N**
3C					C6540U	-	C6540U	-
					C6588U	-	C6588U	-
**3D**					**A7396G**	**T263A**	**A7396G**	**T263A**
					C7605U/C	-	C7605U/C	-

a The sequence of the entire viral genome was determined for FMDV M296I after 2, 5, 8 and 10 passages in the presence of 5 mM ribavirin. The populations are those described in Fig. 1B. Mutation and deduced amino acid substitutions are relative to the sequence of the parental clone FMDV M296I.

b FMDV genomic region analyzed; residue numbering is according to [74].

c Mutations and deduced amino acid substitutions found. Amino acid residues (single-letter code) are numbered individually for each protein from the N- to the C-terminus. Boldface type indicates a change in the amino acid residue. Two residues separated by a bar indicate a mixture of two nucleotides in the population, according to the sequence band pattern. Procedures for nucleotide sequencing and identification of FMDV genomic regions are described in Materials and Methods.

### Extinction of FMDV M296I by a combination of 5-fluorouracil and guanidine hydrochloride requires exceeding a minimal mutagenic activity

**Figure 2:**
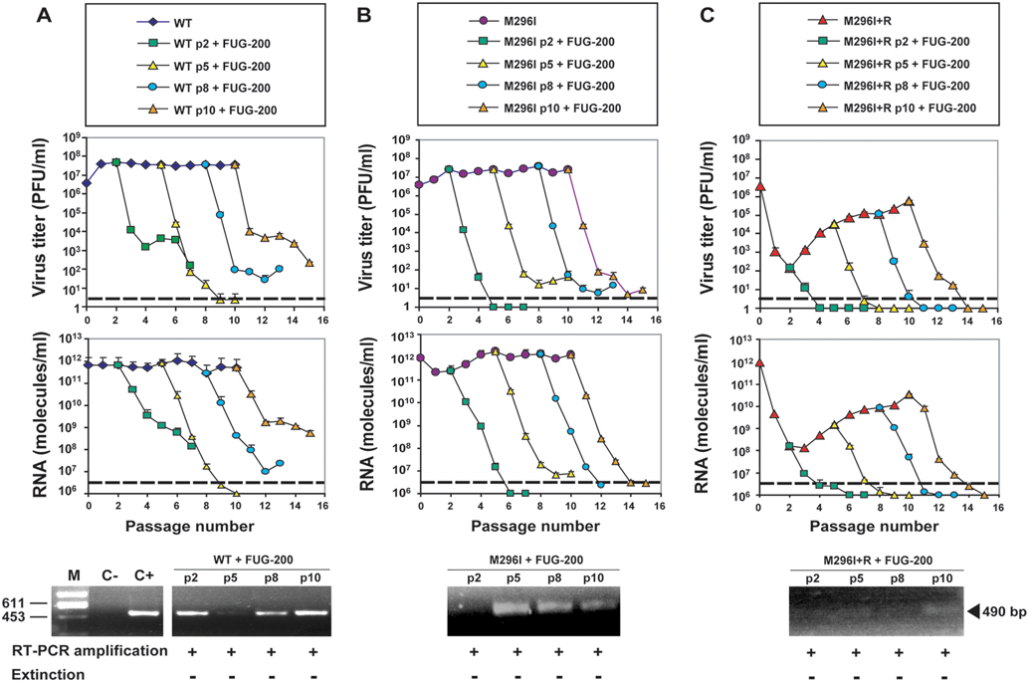
Response of FMDV wild type and mutant M296I to 5-fluorouracil-guanidine combination treatment. FMDV wild type (WT) or mutant M296I were serially passaged in the absence or presence of R (5 mM), as described in the legend for Fig. 1, and as indicated in the boxes at the top. At passages 2, 5, 8 and 10, the viral populations were subjected to additional passages in the presence of a combination of 5-fluorouracil (FU, 200 µg/ml) and guanidinium hydrochloride (GU, 4 mM) (combination FUG-200); p indicates passage number, either in the absence of any drug (panels A and B), or in the presence of 5 mM R (panel C), prior to the FUG-200 treatment. Virus titers and amounts of FMDV RNA in the cell culture supernatants were determined at each passage, as indicated in each panel. Discontinuous lines indicate the limit of detection of infectivity or FMDV RNA. Titrations and quantifications of FMDV were carried out in triplicate; standard deviations are given. Viral RNA was amplified by RT-PCR as a test for virus extinction, as described in Materials and Methods [bottom panels: M, molecular size markers (*Hind* III-digested Ø29 DNA; fragment size in base pairs is indicated on the right); C−, negative control, amplification without RNA; C+, positive RT-PCR amplification control, M, C− and C+ were run for each analysis in A, B, C, but included here in A for simplicity]. Procedures for drug treatments, titration of infectivity, quantification of FMDV RNA by real time PCR, and for RT-PCR amplification are detailed in Materials and Methods.

Since the presence of 3D replacement M296I in FMDV permitted the virus to escape extinction, it was interesting to investigate whether extinction could be achieved by treatment with a different mutagenic agent. Because of previous work with FMDV, FU was the mutagen of choice [46], [48], [49], [54]. However, FU alone was not sufficient to achieve systematic FMDV extinction [46], [48], and, therefore, a combination of FU (200 µg/ml) and GU (4 mM) (termed combination FUG-200) was used [48]. The effect of FUG-200 was tested with FMDV WT and mutant M296I at different passages, and for M296I it was tested also following treatment with R (5 mM). In all cases, the FUG-200 treatment led to a several orders of magnitude decrease in viral infectivity and viral load, that reached values below or close to the limits of detection (Fig. 2). Despite such sharp decreases, FMDV-specific RT-PCR amplifiable material was detected in all cases in the supernatant of the treated, infected cells. A very minor (but still visible) amplification band was discerned in samples of M296I replicated in the presence of R, and then treated with FUG (compare bottom panels in Fig. 2). Thus, despite dramatic decreases of infectivity and viral load, FMDV WT and M296I could not be considered extinguished, according to our criteria of extinction (described in Materials and Methods).

**Figure 3:**
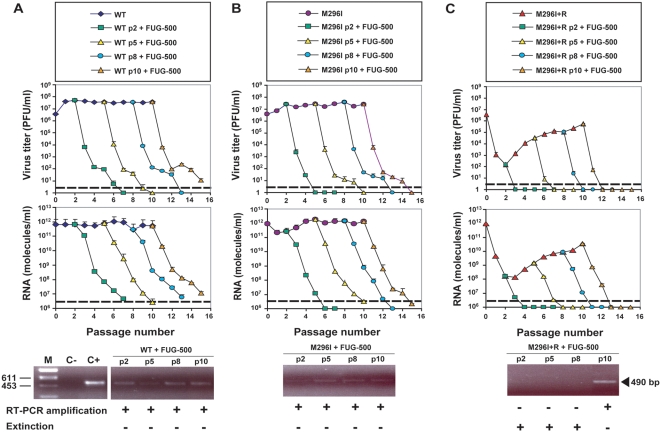
Response of FMDV wild type and mutant M296I to 5-fluorouracil-guanidine treatment, with increased mutagen dose. The experimental design, symbols, drugs, procedures and controls are the same as described in the legend for Fig. 2, except that the FU concentration used was 500 µg/ml FU [FU (500 µg/ml) and GU (4 mM), abbreviated as FUG-500 in the boxes on the top]. Note the absence of infectivity and of an RT-PCR amplification band upon replication of M296I first in the presence of R (5 mM) (2 to 8 passages, but not 10 passages), followed by treatment with FU-500. Extinction of M296I (panel C) was further ascertained by the absence of infectivity or RT-PCR amplifiable viral materials, after three passages of the cells that harbored the putatively extinguished FMDV, in the absence of any drug, as justified in Materials and Methods. Titrations and quantifications of FMDV were carried out in triplicate; standard deviations are given.

FU levels of 100 to 2000 µg/ml do not adversely affect viability of BHK-21 cells under the conditions used for FMDV infection (80% viable cells after 36 h exposure to 2000 µg/ml FU [46]). To test whether increased mutagenic activity could drive FMDV WT and M296I to extinction, we tested a FUG combination with 500 µg/ml FU instead of 200 µg/ml FU, and 4 mM GU, using an identical experimental design as that used with 200 µg/ml FU. This new FUG combination is termed FUG-500. In this case, all populations (except FMDV WT at passage 10) reached infectivity levels below the limit of detection (Fig. 3). The RT-PCR amplification of samples of cell culture supernatants provided amplification bands with minimal intensity for FMDV WT and M296I subjected to FUG-500 treatment, confirming the drastic effect that the treatment had on viral load. Interestingly, no DNA amplification band was detected in the supernatant of cultures infected with M296I in the presence of R for 2, 5 and 8 passages, and then with the FUG-500 combination (compare bottom panels in Fig. 3). To further ascertain extinction, the cells from the cultures that did not show FMDV infectivity and RNA following the FUG-500 treatment, were passaged in the absence of any drug. No infectivity or FMDV RNA were detected in cell culture supernatants, following the extinction test described in Materials and Methods. In contrast to extinction by FUG-500 of the populations passaged 2, 5 and 8 times in the presence of R, no extinction was achieved in the case of the population passaged 10 times in the presence of R (Fig. 3C). Absence of extinction in this case was probably due to the passage history in the presence of R, and to the high fitness attained by M296I after 10 passages in the presence of R [55], [56].

Therefore, a sequential treatment of a FMDV mutant that harbors a R-resistance mutation in its polymerase, first with high R concentrations, and then with a combination of an unrelated mutagen and an inhibitor of viral replication, can result in virus extinction.

### Passage history influences extinction

**Figure 4:**
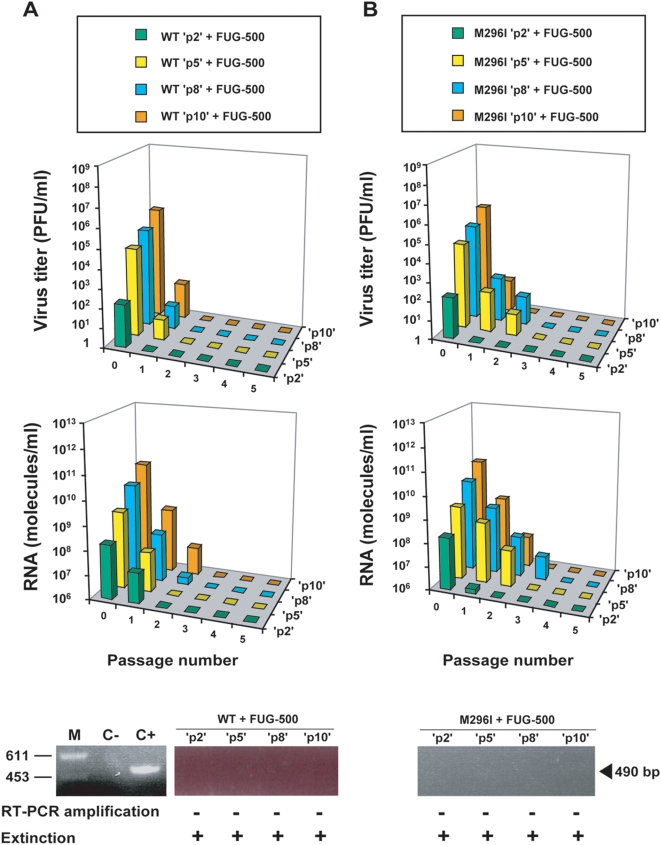
Response of FMDV wild type and mutant M296I to 5-fluorouracil-guanidine combination treatment. Unpassaged FMDV wild type (WT) or mutant M296I was adjusted to a number of PFU/ml comprised between 5.5×10^5^ and 1.5×10^2^ which correspond to the maximum titer (passage 10 in Fig. 3C) and the minimum titer (passage 2 in Fig. 3C) attained by M296I in the presence of R. Subsequently, populations were subjected to passages in the presence of 5-fluorouracil (FU, 500 µg/ml) and guanidinium hydrochloride (GU, 4 mM) (combination FUG-500). The virus (WT, panel A; M296I, panel B) and drug treatment are indicated in the boxes at the top; ‘p2’, ‘p5’, ‘p8’ and ‘p10’ indicate that the number of initial PFU/ml of FMDV WT and M296I was adjusted to the same titer attained by M296I in the presence of R at passage 2, 5, 8 and 10, respectively (data of Fig. 3C). Virus titers and amounts of FMDV RNA in the cell culture supernatants were determined at each passage, as indicated in each panel; titrations and quantifications of FMDV were carried out in triplicate; standard deviations (not given) never exceeded 30% of the mean. Viral RNA was amplified by RT-PCR as a test for virus extinction, as described in Materials and Methods [bottom panels: M, molecular size markers (*Hind* III-digested Ø29 DNA; fragment size in base pairs is indicated on the right); C−, negative control, amplification without RNA; C+, positive RT-PCR amplification control, M, C− and C+ were run for each analysis in A and, B, but included here only in A for simplicity]. Procedures for drug treatments, titration of infectivity, quantification of FMDV RNA by real time PCR, and for RT-PCR amplification are detailed in Materials and Methods.

FMDV M296I passaged in the presence of R had a history of R mutagenesis prior to FUG-500 treatment. Since viral load affects extinction [46], we studied the effect of FUG-500 on M296I, without the counfounding effects of virus passage in the presence of R. To this aim unpassaged FMDV M296I was adjusted to a number of plaque-forming-units (PFU)/ml comprised between 1.5×10^2^ and 5.5×10^5^ which correspond to the range of titers attained by M296I in the presence of R (Fig. 3C). In parallel we also tested FMDV WT with the same range of initial titers. When these unpassaged WT and M296I populations were subjected to FUG-500, extinction was observed in all cases, as evidenced by loss of FMDV infectivity, absence of FMDV RNA, and absence of FMDV-specific RT-PCR amplifiable sequences (Fig. 4). Therefore, passage in BHK-21 cells in the presence of R contributed to extinction-escape of FMDV M296I.

### Extinction-escape is prompted by guanidine-resistance mutations in FMDV

In previous studies on extinction of FMDV by FU and FU-GU combinations, extinction failure was associated with selection of GU-resistant mutants harboring amino acid replacements in non-structural protein 2C [49]. To investigate whether GU-resistance mutations were selected in FMDV WT or M296I populations subjected to 5 passages under FUG-200 or FUG-500, the 2C-coding region was sequenced. Some RNAs were not analyzed because of insufficient amount of material amplified by RT-PCR; they correspond to viral populations that attained very low infectivity and viral RNA levels. The results (Tables 2 and 3) indicate that in all populations subjected to FUG-200 or FUG-500, whose RNA could be amplified, mutations that were previously associated with GU-resistance were present, either alone or with additional mutations (Tables 2 and 3). In particular, substitutions K169R and C141S in 2C, which were dominant in several populations, confer GU resistance in FMDV [49], [50]. I248N was found in 3 populations and, although this substitution had not been previously identified in FMDV populations, I248T was frequently associated with GU resistance [49]. For several populations the region around the nucleotide encoding M296 of 3D was sequenced. For mutant M296I, this substitution was maintained upon FUG treatment, as expected.

**Table 2:** Mutations in FMDV populations subjected to mutagenesis (FUG-200 combination).

FMDV population^a^	Region analyzed (genomic residues)^b^	RT-PCR amplification^c^	Mutation^d^	Amino acid substitution^d^
WT p2	2C (4345–5299)	+	A4850G	K169R
WT p5	2C (4325–5325)	−	N. A.	N. A.
WT p8	2C (4325–5325)	+	A4850G	K169R
WT p10	2C (4326–5308)	+	A4597G	I85V
			A4850G	K169R
M296I p2	2C (4325–5325)	−	N. A.	N. A.
M296I p5	2C (4325–5325)	+	U4765A	C141S
			U5087C	I248N
M296I p8	2C (4327–5299)	+	A4744A/G	R134R/G
			A4850A/G	K169K/R
			U5087U/C	I248I/N
M296I p10	2C (4325–5325)	+	A4850G	K169R
			U5087C	I248N
M296I+R p2	2C (4345–5299)	−	N. A.	N. A.
M296I+R p5	2C (4345–5299)	−	N. A.	N. A.
M296I+R p8	2C (4345–5299)	−	N. A.	N. A.
M296I+R p10	2C (4345–5299)	−	N. A.	N. A.

a The populations correspond to those described in Figure 2 [either WT or M296I subjected to 2, 5, 8 or 10 passages in absence or presence of R 5 mM] and then, in all cases, subjected to 5 passages in the presence of [FU(200 µg/ml)+GU (4 mM); FUG-200 combination]; +R means that passages were carried out in the presence of 5 mM ribavirin.

b The FMDV genomic region analyzed. In all cases, the region that was sequenced spanned the entire 2C-coding region; residue numbering is according to [74].

c It indicates whether the RT-PCR amplification of the genomic region given in the second column produced sufficient DNA for sequencing (+) or not (−). The primers used for the RT-PCR amplification, and the conditions for amplification are detailed in Materials and Methods.

d Mutations and deduced amino acid substitutions found. Two residues separated by a bar indicate a mixture of two nucleotides (and deduced amino acids) in the population, according to the sequencing band pattern. N.A. means not analyzed. Procedures for nucleotide sequencing and identification of FMDV genomic regions are described in Materials and Methods.

**Table 3:** Mutations in FMDV populations subjected to mutagenesis (FUG-500 combination).

FMDV population^a^	Region analyzed^b^ (genomic residues)	RT-PCR amplification^c^	Mutation^d^	Amino acid substitution^d^
WT p2	2C (4328–5323)	+	A4850G	K169R
WT p5	2C (4345–5323)	+	A4850G	K169R
WT p8	2C (4345–5323)	+	A4850G	K169R
WT p10	2C (4331–5319)	+	A4597G	I85V
			A4850G	K169R
M296I p2	2C (4345–5311)	+	A4850G	K169R
M296I p5	2C (4327–5323)	+	A4850G	K169R
M296I p8	2C (4327–5322)	+	A4850G	K169R
M296I p10	2C (4345–5299)	−	N. A.	N. A.
M296I+R p2	2C (4345–5299)	− (RNA extinct)	N. A.	N. A.
M296I+R p5	2C (4345–5299)	− (RNA extinct)	N. A.	N. A.
M296I+R p8	2C (4345–5299)	− (RNA extinct)	N. A.	N. A.
M296I+R p10	2C (4345–5322)	+	No mutations	No substitutions

a The populations correspond to those described in Figure 2 [either WT or M296I subjected to 2, 6, 8 or 10 passages in absence or presence of R 5 mM] and then, in all cases, subjected to 5 passages in the presence of [FU (500 µg/ml) + GU (4 mM); FUG-500 combination]; +R means that passages were carried out in the presence of 5 mM ribavirin.

b The FMDV genomic region analyzed. In all cases, the region that was sequenced spanned the entire 2C-coding region; residue numbering is according to [74].

c It indicates whether the RT-PCR amplification of the genomic region given in the second column produced sufficient DNA for sequencing (+) or not (−). The primers used for the RT-PCR amplification, and the conditions for amplification are detailed in Materials and Methods.

d Mutations and deduced amino acid substitutions found. N.A. means not analyzed. Procedures for nucleotide sequencing and identification of FMDV genomic regions are described in Materials and Methods.

In conclusion, the comparative analysis of FMDV WT and mutant M296I populations subjected to R and FU mutagenesis indicates that the presence of a mutagen-resistance mutation can impede viral extinction by increased doses of the same mutagen. Subsequent treatment with a different mutagen-inhibitor combination can lead to virus extinction.

## Discussion

Selection of inhibitor-resistant viral mutants is a consequence of quasispecies dynamics, and an important problem in antiviral therapy. When highly variable RNA viruses attain large viral loads in infected patients, combination therapy with at least 3 inhibitors that target different steps in the virus life cycle has been recognised as a means to prevent or delay selection of inhibitor-resistant mutants. The benefits of combination therapy to counteract quasispecies adaptability have been supported by both, theoretical studies on virus population dynamics, and clinical observations ([57]–[63], among other studies). Mutation frequencies among components of viral quasispecies *in vivo* are such that mutations that confer resistance to antiviral agents are often represented in patients not subjected to therapy with the relevant inhibitors. This includes patients who could not have been infected by a virus harboring the corresponding resistance mutation [64]–[68].

Lethal mutagenesis of viruses has opened prospects for an alternative antiviral strategy consisting in the use of mutagenic agents specific for virus polymerases, with the objective of driving viruses across an extinction threshold [2], [11], [13], [14], [16], [17], [69], [70]. Experimental, theoretical and *in silico* studies, suggest that one of the molecular mechanisms that contribute to virus extinction by lethal mutagenesis is interference by a subclass of mutant viral genomes, termed defectors [71]–[73]. According to this model, interference by defectors generated under enhanced mutagenesis is added to the increased proportion of genomes with lethal mutations that favor extinction through a reduction of viral load [46]. With these underlying influences, it is not clear what the effect of a mutation that decreases partially the sensitivity to a mutagenic agent would be regarding extinction.

The evidence that R is mutagenic for FMDV includes (i) increase of mutation frequency of mutant spectra of populations passaged in the presence of R, and a dominance of C→U and G→A transitions, relative to populations passaged in the absence of R [18], [37]; (ii) selection of a FMDV mutant that encodes substitution M296I in the viral polymerase (3D) in populations passaged in the presence of increasing concentrations of R, but not in populations passaged in parallel but in the absence of R [37]. Replacement M296I in 3D has not been detected in mutant spectra from FMDV populations not subjected to mutagenesis, or subjected to mutagenesis by FU or 5-azacytidine or subjected to plaque-to-plaque transfers [46], [48], [49], [53], [74]–[76] (iii) FMDV encoding M296I in 3D displayed increased fitness relative to the wild type virus in the presence of R but not in its absence [37]. (iv) In assays carried out with purified 3D in vitro, the enzyme with M296I showed decreased incorporation of RTP oposite C or U in the template, relative to the wild type enzyme. These results indicate that R is mutagenic for FMDV, that substitution M296I was selected specifically upon replication of FMDV in the presence of R, and that M296I conferred a selective advantage to the virus when replicating in the presence of R but not in its absence. Both, virological and biochemical analyses did not provide any evidence that M296I increased the fidelity of the viral polymerase. In fact, a modest decrease in copying fidelity was observed. The reason why FMDV mutant in not extinguished by R is probably that substitution M296I specifically decreases the incorporation of RMP during RNA synthesis [37], [45]. The modest decrease in fidelity in unlikely to provide a number of defectors genomes sufficient to outweigh the decrease of defector genomes due to R-mediated mutagenesis during replication of the mutant virus.

The mechanism by which M296I in 3D of FMDV confers resistance to R is different from the mechanism by which substitution G64S of PV3D exerts its resistance. M296I is located at the β9-α11 loop of FMDV 3D which is critical for nucleotide recognition at the active site of the enzyme [77], [78]. This substitution specifically decreases the capacity of the enzyme to use RTP as substrate. In contrast, G64S in 3D of PV is away from the catalytic site of the polymerase and, through distant interactions, it increases the copying fidelity of the polymerase [39]–[42]. These studies with mutant 3D of FMDV and PV indicate that picornaviruses have at least two mechanisms to render the virus less sensitive to ribavirin: (i) a general increase of fidelity that decreases incorporation of incorrect nucleotides, and (ii) a change that specifically decreases the incorporation of RTP (reviewed in [79]).

FMDV is about 10-fold more sensitive to R in the course of persistent infections than during cytolytic infectious in the same BHK-21 cells [18], [80]. This permits sustained replication of cytolytic FMDV over several passages in the presence of elevated concentrations of R, thus allowing evaluation of the effects of genetic and environmental conditions on the kinetics of extinction. As described in Materials and Methods, the presence of 5 mM R for 31 hours in BHK-21 cell monolayers under the conditions used for infections with FMDV, allowed survival of 40% to 50% of cells. Therefore, toxicity for cells was not the reason for FMDV extinction, as further supported by survival and increased replication of FMDV mutant M296I upon passage in the presence of 5 mM R (Fig. 1B).

In the present report we have addressed this question by investigating whether a mutation that decreases sensitivity of FMDV to R can impede extinction of the virus in the course of cytolytic passages of the virus. The results have shown that replication of M296I and WT FMDV follow different trajectories when replicating in the presence of 5 mM R, and replacement M296I prevented virus extinction. Interestingly, however, in the course of passage of FMDV WT in the presence of 5 mM R, no mutant with M296I in 3D became dominant, and the virus was extinguished (Fig. 1). It must be emphasized that the initial WT and mutant viruses differed exclusively in substitution M296I since they were reconstructed from infectious plasmids, as described in Materials and Methods. New mutations arise continuously upon FMDV replication in cell culture both in the absence and the presence of R [18], [37], [46], [81]. The requirement of initially low and then gradually increasing R concentrations to select for mutant M296I [37], and the failure to select the same mutant by subjecting the same virus to 5 mM R, may relate to suppressive effects of the FMDV quasispecies when populated by higher frequencies of mutant genomes generated under elevated mutagenic intensities [71], [73]. An alternative, not mutually exclusive possibility, is that as the multiplicity of infection (MOI) decreases with the passage number, a potential R-resistant mutant that is generated with low frequency is excluded from the following passage. Experiments are in progress to test these possibilities. Two conclusions can be derived from these experiments: (i) once established as dominant in a viral population, a mutagen-resistant mutation can prevent viral extinction by high doses of the same mutagenic agent; (ii) a wild type virus need not select a mutagen-resistance mutation, when replicating under high concentrations of the mutagen, and, therefore, extinction may occur.

As an approach to drive FMDV mutant M296I towards extinction we studied the fate of WT and mutant populations subjected to mutagenesis with a combination of FU and GU. We had previously established that a combination of FU and GU was more efficient than FU alone in driving FMDV towards extinction [48], [49]. Therefore, we chose to use the FUG combination as a means to extinguish M296I, the latter either with or without a history of R mutagenesis. In the two FUG regimes tested (with either 200 µg/ml or 500 µg/ml FU), the treatment was successful in that very low or undetectable infectivity and viral load of WT and M296I FMDV were achieved. There was no evidence that the presence of mutation M296I impaired the antiviral activity of FUG (compare panels A and B in Figs. 2 and 3). Yet, when a more stringent extinction criterion was used (absence of viral genetic material amplifiable by RT-PCR), extinction was ascertained only for M296I treated with R and then with FUG (with 500 µg/ml FU, not with 200 µg/ml FU) (compare panels C of Figs. 2 and 3). Therefore, extinction of M296I was favored by prior R mutagenesis, and a minimum mutagenic dose by FU was required for extinction. Extinction failure was associated with the presence of GU-resistant replacements in 2C. This results confirms and extends previous observations [49], and alerts of the problem to render an inhibitor inefficient when administered together with a mutagen that enhances the mutation rate.

We cannot exclude that treatment with a combination of the two mutagens FU and R could also result in virus extinction, and these experiments are now in progress. Here, important considerations are toxicity effects derived from the administration of two mutagens, their doses, and confounding effects that might ensue from the simultaneous action of two mutagenic agents that tend to produce opposite mutation types (mainly G→A and C→U by R, versus mainly A→G and U→C by FU [18], [37], [45], [46], [54]). Preliminary experiments show that passage of mutant M296I in the presence of FU alone tends to revert this substitution since reversion depends on a A→G transition, a type of mutation favored by FU treatment [37], [54] (M. Sierra and E. Domingo, unpublished results). Thus, R and FU exert opposed selective pressures regarding the genetic stability of mutant M296I. Also, most mutations that have been associated with resistance of FMDV to GU are A→G transitions, and they could have been favored by the presence of FU in the FUG combination.

Concerning the interpretation of our results and a possible clinical application of lethal mutagenesis, the effects of FU and R on the host cells and organisms must be taken into consideration. FU derivatives can act as inhibitors of enzymes involved in nucleotide metabolism, and can be incorporated into DNA and all forms of RNA, affecting gene expression ([18], [48], [49], [82], [83], reviewed in [18]). R can have several effects on nucleotide metabolism, and as immunomodulator [26], [27], [84]. Ribavirin monophosphate (RMP) is a competitive inhibitor of inosine monophosphate dehydrogenase (IMPDH) thereby lowering the levels of intracellular GTP. Such reduction can contribute to some mutagenic activity due to imbalances in the ribonucleotide pool concentrations [18]. As a G analogue, R can inhibit m-RNA capping and the activity of RNA polymerases [85]. In a microarray-based study of the effect of R on epithelial cells infected with respiratory syncytial virus, R potentiated the virus-induced IFN-stimulated response element (ISRE) that enhances the expression of antiviral response genes stimulated by IFN [86]. These effects of R suggest a mechanism to explain the efficacy of a combination treatment involving R and IFN-α in chronic viral infections, such as HCV infections [86], [87]. Thus, the current evidence is that R exerts its antiviral activity through a number of alternative and not mutually exclusive mechanisms. These multiple influences on the host will require an adequate dosing of FU and R as antiviral agents, and careful consideration of possible inhibitors to be included in the formulations.

Estimates of the R concentration reached *in viv*o during antiviral treatments are of 600–800 µM at the site of infection after aerosol administration, and maximum serum or cerebrospinal fluid concentrations of 20–150 µM after intravenous or oral administration ([88]–[90]; reviewed in [18]). These concentrations are lower than those used in our model studies. Extinction as a direct result of the treatment, which in our experiments has been taken as the most strict test of antiviral efficacy, may not be necessary to clear virus *in vivo*. A decrease in viral load may be sufficient to give an opportunity to the immune system to clear the virus. Also, early inititation of treatment, before a virus increases its fitness in the infected organism, will be more effective to reduce the viral load and to eliminates the virus.

In conclusion, FMDV populations doomed to extinction failure because of the dominance of a mutagen-resistant mutation, could still be driven towards extinction by a combination treatment involving another mutagen and a non-mutagenic antiviral inhibitor. We are currently planning the exploration of this lethal mutagenesis protocol *in vivo*, using a mouse model for FMDV, a system previously used to document that the mutant spectrum composition of the viral quasispecies plays an essential role in virulence [91]. Possible lethal mutagenesis protocols suggested by the present study avoid the simultaneous administration of two mutagenic gents, thereby minimizing toxicity for cells or organisms.

## Materials and Methods

### Cells and viruses

The origin of BHK-21 cells and procedures for cell growth in Dulbecco's modification of Eagle's medium (DMEM), and for plaque assays in semisolid agar have been previously described [81], [92]. To control for the absence of contamination, supernatants of mock-infected cells were titrated in parallel; no signs of infectivity or cytopathology in the cultures or in the control plaque assays were observed in any of the experiments.

### Mutagenic treatments

A solution of ribavirin (R) in PBS was prepared at a concentration of 100 mM, sterilized by filtration, and stored at −70°C. Prior to use, the stock solution was diluted in DMEM to reach the desired R concentrations. For infections in the presence of R, cell monolayers were treated during 7 h with 5 mM R prior to infection. After adsorption of FMDV and washing of the cell monolayers, the infection was allowed to proceed in the presence of same concentration of R. FMDV C-S8c1 was passaged serially in the absence or in the presence of R (5 mM). For each passage 2×10^6^ BHK-21 cells were infected with supernatant of virus from the previous passage (0.2 ml), and the infection allowed to proceed for about 24 h. In each passage carried out in the absence of R, 2×10^6^ BHK-21 cells were infected with 9.8×10^6^–1.4×10^6^ PFU of WT or mutant M296I. In the presence of R, 2×10^6^ BHK-21 cells were infected with 2.1×10^4^–2.2×10^2^ PFU (first passage) and 5×10^2^ PFU down to 1 PFU (for WT populations that reach very low infectivity). Values for each passage can be estimated from the infectivities given in Figure 1. Infections in the absence of R, and mock-infected cells, were maintained in parallel; no evidence of contamination of cells with virus was observed at any time. Incubation of confluent BHK-21 cells monolayers (as used for infections with FMDV) with 5 mM R for 31 hours resulted in cell viabilities of 40% to 50%, as determined with trypan blue exclusion (range calculated in six different determinations). Therefore, toxicity of R for the cells cannot account for FMDV extinction (see also Discussion).

To prepare culture medium containing 5-fluorouracil (FU) (Sigma), the analogue was dissolved in DMEM to yield a 5 mg/ml solution, and diluted in DMEM, as needed for the experiments. To prepare medium containing guanidine (GU) (Sigma), a solution of GU in DMEM was prepared at a concentration of 50 mM. To prepare medium containing a mixture of both FU and GU, the appropriate amount of GU was added to DMEM containing FU to yield solutions containing either FU (200 µg/ml) and GU (4 mM), termed FUG-200, or FUG (500 µg/ml) and GU (4 mM), termed FUG-500. All solutions were sterilized by filtration. Precautions to ensure stability of the drug preparations prior to use, and measurements of toxicity for BHK-21 cells, and have been previously described [18], [46].

For infections in the presence of FU and GU, confluent cell monolayers were pretreated for 13 h with 200 µg/ml or 500 µg/ml FU. After virus adsorption, the infections were allowed to proceed in the presence of FUG-200 or FUG-500 for approximately 24 h. The inhibition of infectious progeny production associated with the different treatments described in the present study, in a standard FMDV infection at a MOI of 1–2 PFU/cell was: 99.7% for treatment with 5 mM R, and 99.9% for treatment with the FUG-200 or FUG-500 combinations. For serial passage of virus, 0.2 ml of cell supernatant from the previous infection was used to infect a cell monolayer (2×10^6^ BHK-21 cells) pretreated with FU as described above. In the course of treatment with FUG-200 or FUG-500 of viruses not exposed to R, 2×10^6^ BHK-21 cells were infected with 5×10^6^–9×10^6^ PFU (first passage) and 2×10^3^–5×10^3^ down to 1 PFU (for populations that reach very low infectivity). For M296I treated with R, 2×10^6^ BHK-21 cells were infected with 1.1×10^5^–1.5×10^2^ PFU (first passage) and 6.2×10^2^ down to 1 PFU (for populations that reach very low infectivity). Values for each passage can be estimated from the infectivities given in Figures 2 and 3. No FMDV extinction occurs in serial viral passages carried out at very low MOI in the absence of mutagens [48], [49]. Virus was titrated after each passage. All titrations were carried out in triplicate, and standard deviations calculated, as indicated in the corresponding experiments. Mock-infected cells were maintained in parallel; no evidence of contamination of cells with virus was observed at any time.

### RNA extraction, RNA quantification, cDNA synthesis and PCR amplification

Viral RNA was extracted from the medium of infected cultures using Trizol (Invitrogen) as previously described [53]. Reverse transcription was performed with AMV reverse transcriptase (Promega), and PCR amplification was carried out using Expand High Fidelity (Roche), as specified by the manufacturers. The 2C-coding region was amplified using as primers oligonucleotides 2BR2 (5′-GAAGACCTTGAGAGAGCAG; sense orientation; the 5′ nucleotide corresponds to genomic residue 4318), and 2CD4 (5′- ACACAGATTTTTGGGAAGGT; antisense orientation; the 5′ nucleotide corresponds to genomic residue 5326). The 3D-coding region was amplified using two sets of primers. One set included oligonucleotides A2SacI (5′-CACACATCGACCCTGAACCGCACCACGA; sense orientation; the 5′ nucleotide corresponds to genomic residue 6581), and AV4 (5′- TTCTCTTTTCTCCATGAGCTT; antisense orientation; the 5′ nucleotide corresponds to genomic residue 7071). The second set included oligonucleotides 3DR1 (5′- GAAACGCCGCGGTGCACTTATC; sense orientation; the 5′ nucleotide corresponds to genomic residue 6984), and ED6 (5′-AATCATTGTGCCAATTGTGAT; antisense orientation; the 5′ nucleotide corresponds to genomic residue 8053). Genomic residues are numbered as described in [74]. Amplification products were analyzed by agarose gel electrophoresis using *Hind* III-digested Ø-29 DNA as molar mass standards. Negative controls (amplifications in the absence of RNA) were included in parallel to ascertain absence of contamination by template nucleic acids. No evidence of contamination with extraneous templates was obtained in any of the experiments reported here.

### Assessment of FMDV extinction

FMDV was considered extinct when no virus infectivity and no viral RNA that could be amplified by a highly sensitive RT-PCR protocol, could be demonstrated neither in the supernatant of the passage that harbors the putatively extinguished virus, nor after 3 blind passages in BHK-21 cells, in the absence of any drug. Multiple highly sensitive RT-PCR amplification reactions that yield short cDNAs were carried out to ascertain extinction. These criteria to consider FMDV extinct [18], [37], [46], [48], [49] have now been extended to show that no infectivity or RT-PCR amplifiable material can be retrieved after three passages of the cells that harbor the putatively extinguished virus. This extension was prompted by the observation that FMDV subjected to hundreds of plaque-to-plaque transfers could lose capacity to form plaques and yet maintain intracellular RNA [53]. It should be noted the loss of infectivity below the level of detection did not necessarily imply extinction of viral genetic material (compare Figs. 2 and 3 in Results).

### Quantification of FMDV RNA

Real time quantitative RT-PCR was carried out using the Light Cycler RNA Master SYBR Green I kit (Roche), according to the instructions of the manufacturer, as described previously for FMDV RNA [53]. The 2C-coding region was amplified using as primers oligonucleotides 2CR2 (5′- GGCAAACCCTTCAGCAGTAAG; sense orientation; the 5′ nucleotide corresponds to genomic residue 4924), and 2CD3 (5′- CGCTCACGTCGATGTCAAAGTG; antisense orientation; the 5′ nucleotide corresponds to genomic residue 5047). Quantification was relative to a standard curve obtained with known amounts of FMDV RNA, synthesized by *in vitro* transcription of FMDV cDNA (plasmid pMT28, described in [93]). The specificity of the reaction was monitored by determining the denaturation curve of the amplified DNAs. Negative controls (without template RNA, or with RNA from mock-infected cells) were run in parallel with each amplification reaction. Again, no evidence of contamination with extraneous templates was obtained in any of the experiments reported here.
